# Prospective evaluation of glutamine and phospholipids levels in first degree relatives of patients with Type 1 Diabetes from a multiethnic population

**DOI:** 10.1186/s13098-015-0048-x

**Published:** 2015-06-10

**Authors:** D.B. Araujo de Pina Cabral, J.R. Dantas, H. Skärstrand, B. Barone, F. Carvalho, R. Tortora, A. Milech, F. Vaziri-Sani, J.E. P. Oliveira, L. Zajdenverg, M. Rodacki

**Affiliations:** Department of Nutrology, Federal University of Rio de Janeiro, Avenida Epitácio Pessoa, número 2.990, apto 806, Lagoa, Rio de Janeiro Brazil; Department of Clinical Sciences, Lund University, Skåne University Hospital (SUS), Malmö, Sweden; Biochemistry Laboratory, Federal University of Rio de Janeiro, Rio de Janeiro, Brazil

**Keywords:** Metabolic, Diabetes, Non-whites, Antibodies, Multiethnic

## Abstract

**Background:**

A dysregulation in the metabolism of lipids may be an early marker of autoimmunity in Type 1 Diabetes (T1D). It would be of general importance to identify metabolic patterns that would predict the risk for T1D later in life. The aim of this study was to perform a prospective evaluation of glutamine and phospholipids levels in Brazilian first degree relatives (FDR) of patients with T1D in a mean interval of 5 years.

**Findings:**

Brazilian FDR (*n* = 30) of patients with T1D were evaluated and blood was sampled to measure the levels of glutamine and phospholipids in the fasting serum by quantitative colorimetric method. The tests were repeated after a mean interval of 5 years and compared to a control group (*n* = 20). The FDR presented lower levels of phospholipids than controls (*p* = 0.028), but not of glutamine (*p* = 0.075). Phospholipids levels decreased over time (*p* = 0.028) in FDR and were associated with Glutamic acid decarboxylase autoantibody (GADA) titers (*p* = 0.045), autoantibody positivity (*p* = 0.008) and PTPN22 polymorphisms (*p* = 0.014).

**Conclusions:**

In this Brazilian multiethnic population, there was a significant decrease in phospholipids levels in FDR in patients with T1D during a 5-year prospective follow-up, as well as a significant association between these metabolite, GADA and PTPN22 polymorphisms. For Glutamine no difference was found. These findings suggest that a dysregulation in the metabolism of lipids may precede the onset of the autoimmunity in T1D.

## Introduction

The autoimmune process in Type 1 Diabetes (T1D) may be a late response to a dysregulation in the metabolism of lipids and amino acids [1–4]. The DIPP (Diabetes Prediction and Prevention Project) and BABYDIAB (a German prospective study from birth on the natural history of type 1 diabetes) studies found reduced levels of methionine and glutamine before islet autoanibody seroconversion in individuals that developed T1D later in life. Oresic et al. also demonstrated decreased levels of phospholipids and triglycerides in the pre-clinical period of T1D [2, 3].

The amino acids and lipids play an important role in regulating β cells insulin secretion. Glutamine, the most abundant amino acid in blood, presents antioxidant properties, protecting β cells from oxidative insults. Besides, phospholipids may also be a key modulator of insulin release. Therefore, it would be of general importance to identify metabolic patterns that would predict the risk for T1D later in life [4–6].

As most investigations in this field have included only Caucasians, very little is known about the natural history of T1D in other ethnic groups, such as the Brazilian population. It is possible that different populations present peculiarities in the development of T1D. Therefore, it is important to identify if the same metabolic alterations occur in non-Caucasian individuals. This was the first study that analyzed the metabolic profile of FDR of patients with T1D in a multiethnic population.

We aimed to perform a prospective evaluation of glutamine and phospholipids levels in Brazilian FDR of patients with T1D in a mean interval of 5 years.

## Methods

### Subjects

First degree relatives (FDR) of Brazilian patients with T1D (*n* = 30, baseline sample) were interviewed and blood was sampled for DNA extraction, metabolites and autoantibody measurement. Participants were classified as whites or non-whites (mostly Afro-descendants) based on their phenotype and family background [7]. The results from metabolites were compared to non-diabetic controls (*n* = 20).

### Measurement of metabolites

The quantitative colorimetric assays for glutamine and phospholipids measurement were performed at the Federal University of Rio de Janeiro, Rio de Janeiro, Brazil. The metabolites were measured in fasting serum of FDR and in control group by quantitative colorimetric method (EnzyChrom Assay Kit of Bioassay Systems). Since the reference range for these tests have not been defined, the frequency of individuals above and below the 50th percentile (p50) obtained in the healthy controls was investigated.

### Measurement of autoantibodies

Antibodies against GAD, insulin and insulinoma-associated antigen-2 (GADA, IAA and IA2A, respectively) were measured by radioimmunoassay (RIA) [8–11].

Serum samples were also analyzed by a standard radioligand binding assay (RBA) for the three individual ZnT8A variants (ZnT8RA, ZnT8WA, ZnT8QA) as well as with the ZnT8TripleA assay, as previously reported [8, 11].

### Genetic analysis

The FDR were genotyped for INS gene and PTPN22 polymorphisms. SNP for INS and PTPN22 (polymorphism RS620W rs2476601) were genotyped with fluorogenic allele-discrimination chemistry [12–14].

### Prospective evaluation

After 5 years (a mean interval of 3.8 to 6.3 years), the measurement of glutamine, phospholipids and autoantibodies was repeated. The same technique described above was used.

### Statistical analysis

Mann–Whitney *U* test and chi-square were used for comparison between groups. Spearman coefficient was used to test correlation between continuous variables, and Fisher’s exact *t*-test to test correlation between categorical variables. A p-value <0.05 was considered significant. The software applied was SPSS for Windows, version 17.0 (SPSS Inc.)

## Results

The general characteristics of the sample are summarized in Table 1.

**Table 1:** General characteristics of the study sample

	Basal	Prospective	Controls	*p* value
	FDR	*FDR*		
	(*n* = 30)	(*n* = 30)	(*n* = 20)	
Age (years)	17.96	17.36	25.60	0.108
(mean ± SD)	±9.24	±8.68	±9.65	
Gender [n (%)]				
Female	15 (50)	15 (50)	9 (45)	0.184
Etnicity[n (%)]				
Whites	15 (50)	15 (50)	8 (40)	0.272
Non-whites	15 (50)	15 (50)	12 (60)	
GADA [n (%)]	2 (7)	2 (7)	2 (10)	0.887
IA2A [n (%)]	2 (7)	1 (3)	0	*----*
IAA [n (%)]	0	1 (3)	0	*----*
ZnT8A [n (%)]	1 (3)	1 (3)	0	0.002
Glutamine (mM) (mean ± SD)	1.45 ± 0.69	1.34 ± 0.20	1.55 ± 0.27	0.075
Phospholipids (μm) (mean ± SD)	505.20 ± 194.89	468.56 ± 219.46	640.00 ± 259.20	0.028

Basal FDR = sample of the basal period; Prospective FDR = sample of the prospective period

Controls = non-diabetic individuals; SD = standard deviation; GADA = Glutamic acid decarboxylase autoantibody; IA2A = Insulinoma-associated antigen-2 autoantibody; IAA = autoantibody against insulin; ZnT8A = Zinc transporter 8 autoantibody

### Glutamine levels

**Fig. 1:**
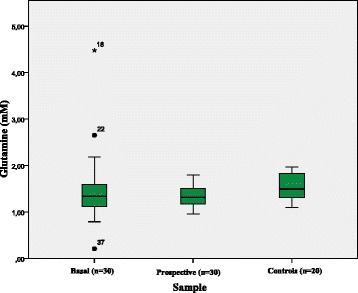
Glutamine levels (mM) in basal sample, prospective sample and controls. The Glutamine levels did not differ between these three groups (*p* = 0.075)

The mean levels of glutamine found in FDR and controls are shown in Table 1. There was no significant variation in the levels of this metabolite in a period of 5 years (*p* = 0.075) (Fig. 1).

The majority of the FDR presented glutamine levels below the 50th percentile in relation to the median of the control group (23 % or 71.9 % in the baseline sample and 26 % or 86.7 % in the prospective sample), but the difference between these groups was not significant (*p* = 0.075). There was no association between glutamine levels and positive titers of antibodies (*p* = 1.000).

### Phospholipids levels

**Fig. 2:**
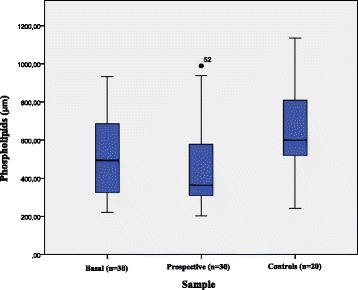
Phospholipids levels (μm) in basal sample, prospective sample and controls. The prospective group showed diminished levels of phospholipids in relation to the basal samples and controls (*p* = 0.028)

The mean levels of phospholipids found in FDR and controls are shown in Table 1. There was a significant variation in the levels of this metabolite in a period of 5 years (*p* = 0.028) (Fig. 2). The majority of the FDR showed phospholipids levels below the 50th percentile in relation to the median of the control group (21 % or 65.6 % in the baseline sample and 24 % or 80 % in the prospective sample), with a significant difference between these groups (*p* = 0.028). There was a significant association between low phospholipids levels, positive titers of GADA (*p* = 0.045) in FDR at baseline, and between positivity for autoantibody (GADA) (*p* = 0.008) in the prospective evaluation.

### Genetic polymorphisms

For PTPN22, the prevalence of homozygous was 93.3 % and heterozygous was 6.7 %. None of the FDR presented homozygous T/T for rs24766001 polymorphism of PTPN22.

In the prospective evaluation, there was a significant association between PTPN22 polymorphisms and low phospholipids levels (*p* = 0.014).

The prevalence of rs689 polymorphism of the insulin gene was: homozygous at 43.3 %, heterozygous 40 % or homozygous at 16.7 % . There were no significant differences between polymorphisms of the insulin gene and autoantibodies or metabolites.

## Discussion and conclusions

In this study we found a significant decrease in phospholipids levels in FDR of patients with T1D during a 5-year prospective follow-up, as well as a significant association between low levels of this metabolite, GADA and PTPN22 polymorphisms. In this study population, most of the FDR presented lower levels of phospholipids in comparison to the control group, similar to the findings in the BABYDIAB cohort. Although the majority of FDR had low glutamine levels, there was no significant difference in comparison with controls. A positive correlation between phospholipids levels, positivity for autoantibody and titers of GADA was found. The altered levels of glutamine, phospholipids, and other metabolites in children at high risk for T1D, before the seroconversion of pancreatic autoantibodies have been repported [2, 3]. This dysregulation in the metabolism of lipids may be an early marker of autoimmunity, but it still remains unclear if these individuals with low phospholipids levels will have a higher risk of developing T1D [4–6].

The presence of the polymorphic C allele was strongly associated with low levels of phospholipids. Previous studies have shown that polymorphisms of this gene increases the risk of autoimmune diseases emergence by acting in the immunoregulation [12–20]. To our knowledge, the association between PTPN22 polymorphisms and metabolites has not been reported in the literature. This finding may represent a peculiarity of our multiethnic population and further studies in other ethnic groups are necessary.

To conclude, our study found reduced levels of metabolites in FDR of T1D patients, and a positive association between phospholipids, GADA and PTPN22. Some of these results may represent uniqueness of our multiethnic population, while others peculiarities may be common to other populations with T1D. These findings could support further understanding of the pathogenesis of T1D, as well as in stratification of individuals at risk of developing T1D and, contributing to prevention strategies.

## Abbreviations

GADA: Glutamic acid decarboxylase autoantibody
IA2A: Islet antigen-2 autoantibody
INS: Insulin
FDR: First degree relatives
T1D: Type 1 Diabetes
ZnT8RA: Arginine 325 Zinc transporter 8 autoantibody
ZnT8WA: Tryptophan 325 Zinc transporter 8 autoantibody
ZnT8QA: Glutamine 325 Zinc transporter 8 autoantibody
ZnT8A: Zinc transporter 8 autoantibody to one, two or all three amino acid variants

## References

[1] Van Belle TL, Coppieters KT (2011). Type 1 diabetes: etiology, immunology, and therapeutic strategies. Physiol Rev.

[2] Oresic M, Simell S (2008). Dysregulation of lipid and amino acid metabolism precedes islet autoimmunity in children who later progress to type 1 diabetes. J Exp Med.

[3] Pflueger M, Seppanen-Laakso T (2011). Age- and islet autoimmunity-associated differences in amino acid and lipid metabolites in children at risk for type 1 diabetes. Diabetes.

[4] Newsholme P, Cruzat V (2014). Nutrient regulation of insulin secretion and action. J Endocrinol.

[5] La Torre D, Seppänen-Laakso T (2013). Decreased cord-blood phospholipids in young age at onset type 1 diabetes. Diabetes.

[6] Cruzat VF, Keane K (2014). Alanyl-glutamine improves pancreatic β-cell function following ex vivo inflammatory change. J Endocrinol.

[7] Palatnik M, Da Silva WA (2002). Ethnicity and type 2 diabetes in Rio de Janeiro, Brazil, with a review of the prevalence of the disease in Amerindians. Hum Biol.

[8] Vaziri-Sani F, Oak S (2010). ZnT8 autoantibody titers in type 1 diabetes patients decline rapidly after clinical onset. Autoimmunity.

[9] Brorsson C, Vaziri-Sani F (2011). Correlations between islet autoantibody specificity and the SLC30A8 genotype with HLA-DQB1 and metabolic control in new onset type 1 diabetes. Autoimmunity.

[10] Andersson C, Larsson K, Vaziri-Sani F (2011). The three ZNT8 autoantibody variants together improve the diagnostic sensitivity of childhood and adolescent type 1 diabetes. Autoimmunity..

[11] Vaziri-Sani F, Delli AJ, Elding-Larsson H, Lindblad B, Carlsson A, Forsander G, Ivarsson SA, Ludvigsson J, Marcus C, Lernmark A (2011). A novel Triplemix RBA. J Immunol Methods. Vol..

[12] Begovich AB, Carlton VEH, Honigberg LA, Schrodi SJ, Chokkalingam AP (2004). A missense singlenucleotide polymorphism in a gene encoding a protein tyrosine phosphatase (PTPN22) is associated with rheumatoid arthritis. Am J Hum Genet..

[13] Bottini N, Vang T, Cucca F, Mustelin T (2006). Role of PTPN22 in type 1 diabetes and other autoimmune diseases. Semin Immunol..

[14] Barone B, Dantas JR, Almeida MH, Anna-Gomes BS, Bencke-Gongalves MDR, Albernaz MS (2011). Pancreatic autoantibodies, HLA DR and PTPN22 polymorphisms in first degree relatives of patients with type 1 diabetes and multiethnic background. Exp Clin Endocrinol Diabetes.

[15] Concannon P, Rich SS, Concannon P, Rich SS (2009). Genetics of type 1A diabetes. N Engl J Med.

[16] Howson JM, Walker NM (2009). Analysis of 19 genes for association with type I diabetes in the Type I Diabetes Genetics Consortium families. Genes Immun..

[17] Karvonen M, Pitkaniemi M (1997). Sex difference in the incidence of insulin-dependent diabetes mellitus: an analysis of the recent epidemiological data. World Health Organization DIAMOND Project Group. Diabetes Metab Rev..

[18] Steck AK, Bugawan TL (2005). Association of non-HLA genes with type 1 diabetes autoimmunity. Diabetes.

[19] Steck AK, Wong R (2012). Effects of non-HLA gene polymorphisms on development of islet autoimmunity and type 1 diabetes in a population with high-risk HLA-DR,DQ genotypes. Diabetes.

[20] Yu J, Yu L (2000). Transient antiislet autoantibodies: infrequent occurrence and lack of association with “genetic” risk factors. J Clin Endocrinol Metab..

